# A Review of Walnut Allergy: Allergens Characteristic, the Impact of Processing on Allergenicity and Future Perspectives

**DOI:** 10.3390/foods15132321

**Published:** 2026-06-30

**Authors:** Jingyuan Jiang, Bingyu Chen, Xinyu Ma, Dai Yan, Ning Li, Hongzhi Liu

**Affiliations:** 1 Key Laboratory of Geriatric Nutrition and Health, Beijing Technology and Business University, Ministry of Education, Beijing 100048, China; jiangjingyuan0927@163.com (J.J.); bychen@btbu.edu.cn (B.C.); eva6299@163.com (X.M.); 2 DSM (China) Ltd., PuDong New Area, Shanghai 201203, China; dai.yan@dsm-firmenich.com; 3 GNC (Shanghai) Food Products Technology Co., Ltd., Shanghai 200335, China; ning.li@gnc.com.cn

**Keywords:** walnut, allergenic proteins, food processing, allergenicity regulation, hypoallergenic foods

## Abstract

(1) Background: As one of the world’s four major nuts, walnuts are rich in nutritional value; however, concerns regarding their allergenicity are becoming increasingly prominent. (2) Scope and Approach: This article provides a systematic review of the nutritional value and allergenicity of walnuts, the composition of major allergenic proteins, and their detection techniques. A particular focus is placed on elucidating the mechanisms by which different processing methods—including heat treatment, ultra-high pressure, ultrasound, low-temperature plasma, enzymatic treatment, and polyphenol modification—affect the structure and allergenicity of walnut allergenic proteins. (3) Key Findings and Conclusions: Current evidence suggests that processing techniques can alter the secondary and tertiary structures of walnut proteins, change the accessibility of linear or conformational epitopes, and reduce their Immunoglobulin E/Immunoglobulin G (IgE/IgG) binding capacity under certain in vitro conditions. Among these, high-temperature and high-pressure treatment, enzymatic hydrolysis, polyphenol modification, and combined processing strategies demonstrate promising potential for reducing walnut protein immunoreactivity. However, structural modifications, reduced antibody-binding capacity, or increased digestibility should not be directly interpreted as definitive evidence of reduced clinical sensitization. This paper summarizes the current status of the development and application of hypoallergenic foods, analyzes the technical challenges and future development directions, and aims to provide a theoretical basis and technical reference for the development of allergenicity-reduced walnut products.

## 1. Introduction

As one of the top varieties among the world’s four major nuts, the walnut is a rich source of nutrients. Walnut kernels are rich in fats, proteins, carbohydrates; minerals such as phosphorus, calcium, iron, and zinc; as well as various vitamins [1]. Numerous studies have confirmed that long-term consumption of walnuts helps improve cognitive function and reduces the risk of cardiovascular disease, depression, dementia, type 2 diabetes, and other conditions [2]. According to statistics from the U.S. Food and Drug Administration (FDA), more than 90% of food allergies are triggered by nine major allergenic foods: tree nuts, shellfish, fish, eggs, soy, peanuts, milk, wheat, and sesame [3]. In the United States, walnuts are considered the most common allergenic nut [4]. Currently, the prevalence of walnut allergies is on the rise, and walnuts have become one of the major food allergens explicitly required to be labeled by the Codex Alimentarius Commission and the regulations of many countries [5]. The reported prevalence of tree nut allergy varies substantially among regions and according to diagnostic criteria. Previous systematic reviews have shown that the prevalence of probable tree nut allergy ranges from 0.05% to 4.9% [6]. Food allergies primarily refer to rapid allergic reactions mediated by immunoglobulin E (IgE). For individuals with walnut allergies, consuming walnuts or walnut products often causes clinical symptoms of varying severity, significantly impacting their quality of life.

Walnut allergy is clinically important because it is often persistent and may cause severe systemic reactions. Symptoms of walnut allergy generally occur in the respiratory tract, gastrointestinal tract, and skin, typically manifesting as nausea, swollen lips, and other signs; in severe cases, they can even lead to anaphylaxis or death [7,8,9]. Shin et al. have pointed out that contributing factors such as exercise, infection, dehydration, and alcohol consumption can exacerbate food-induced allergic reactions [10]. While most food allergies that develop in infancy are outgrown with age, allergies to buckwheat, peanuts, tree nuts, shellfish, and fish are rarely outgrown [11]. Molecular diagnosis has improved the clinical evaluation of walnut allergy. Conventional diagnosis relies on clinical history, skin prick testing, serum walnut-specific IgE, and, when necessary, oral food challenge. In the NUT CRACKER study, specific IgE to *Jug r 1* or *Jug r 4* demonstrated high diagnostic value for distinguishing patients with walnut allergy from individuals who are sensitized to walnuts but tolerate them [4]. Studies have shown that walnuts are one of the leading causes of anaphylactic shock from nut allergies in children and adolescents, accounting for 16% of related cases [12]. Cross-reactivity is another important clinical issue in walnut allergy. Clinical studies have shown that many walnut-allergic patients are also allergic to pecan, whereas pecan allergy rarely occurs in walnut-tolerant patients. Cross-reactivity may also occur between walnut and other tree nuts, including hazelnut, because cupin and prolamin superfamily allergens may share structurally related IgE-binding epitopes [4]. According to reports, Walnut Jug r 6 can also serve as a marker for cross-reactions between walnuts and other Vicilin-containing foods such as hazelnuts, sesame seeds, and pistachios [13]. From a risk management perspective, strict avoidance of walnut-containing foods remains the main strategy for walnut-allergic patients. Therefore, to protect public health, there is an urgent need to implement appropriate measures to prevent walnut allergies, including but not limited to processing methods to reduce the allergenicity of walnuts.

**Figure 1:**
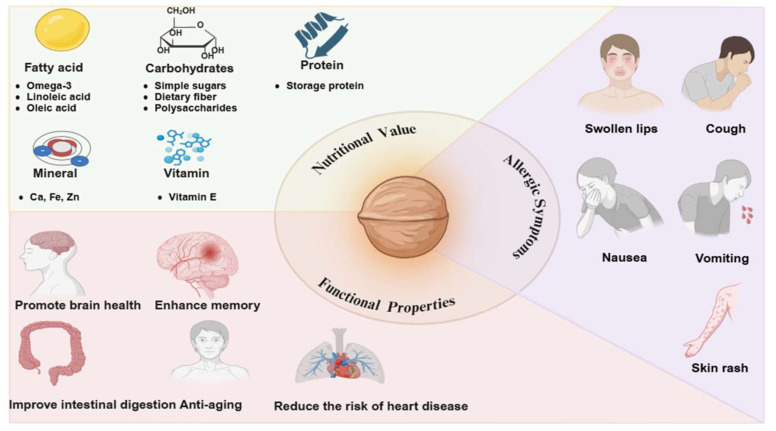
Health Benefits and Allergy Concerns Related to Walnut Protein in the Food Industry.

Reaction thresholds are central to risk assessment. Walnut threshold studies have shown that eliciting doses (ED) vary widely among allergic individuals, and estimated ED values depend on the population and statistical model used. These data are important for allergen risk assessment and precautionary allergen labeling, but they should not be interpreted as universally safe doses for every patient. Although previous reviews have addressed walnut proteins, tree nut allergens, and hypoallergenic processing technologies, these topics are often discussed in isolation. Existing reviews of walnut proteins primarily focus on protein composition, physicochemical properties, functional characteristics, and applications [14], while research on tree nut allergies may emphasize allergen homology, cross-reactivity, diagnosis, and clinical management [4,15,16]. Furthermore, reviews on allergenicity-reducing technologies typically address thermal, non-thermal, enzymatic, or chemical processing methods across a broad range of food systems [17,18], rather than focusing on the major allergens and epitopes of walnuts. Therefore, the innovation of this review lies in its comprehensive viewpoint, which systematically elucidates the major allergens of walnuts and their IgE-binding epitopes, the effects of processing methods on allergen structure and immunoreactivity, relevant clinical evidence, and the feasibility and limitations of industrial applications. This may provide a more systematic foundation for the development, evaluation, and safety assessment of allergenicity-reduced walnut products. Figure 1 illustrates the nutritional value of walnut protein and associated allergic symptoms.

## 2. Composition of Major Walnut Allergenic Proteins and Detection Techniques

### 2.1. Composition of Major Walnut Allergenic Proteins

To date, 11 walnut allergens have been listed on the official website of the Allergen Naming Group of the World Health Organization and the International Union of Immunological Societies (https://www.allergen.org/). These primarily include *Jug r 1* (2S albumin), *Jug r 2* (Vicilin), *Jug r 3* (lipid transfer protein, LTP), *Jug r 4* (Legumin), *Jug r 5* (pathogenesis-related protein 10, PR-10), *Jug r 6* (7S globulin), and *Jug r 7* (profilin) [13,19]. Among these, *Jug r 1*, *Jug r 2*, *Jug r 3*, and *Jug r 4* are considered the most major allergenic proteins.

#### 2.1.1. *Jug r 1*

*Jug r 1* is one of the key allergenic proteins in walnuts and is recognized by the serum of more than 80% of walnut allergy patients [3]. It consists of 139 amino acids and has a molecular weight of approximately 16 kDa [20]. Natural *Jug r 1* consists of two covalently linked polypeptide chains—a light chain (3.5 kDa) and a heavy chain (8 kDa). It is resistant to thermal denaturation and hydrolysis by trypsin and chymotrypsin, but is readily hydrolyzed by pepsin within one hour under acidic conditions. Its secondary structure is dominated by α-helices, followed by random coils, β-turns, and β-sheets, with multiple conformations coexisting [21]. Robotham first precisely localized the key IgE-binding sites of this allergen, discovering a major IgE-binding region on *Jug r 1* [22]. Epitope 1, with the amino acid sequence LKACREVQQVEQG, is located in the N-terminal region exposed on the molecular surface, while epitope 2, with the amino acid sequence DDDDEPRDQQPR, is situated in the central region of the molecule and exhibits acidic properties. Robotham and Sordet et al. jointly confirmed that epitope 3 (QVVRRQQQQ) is the core IgE-binding region of *Jug r 1*. Sordet newly identified epitope 4 (QGLRGEEMEEMV) as adjacent to epitope 3 and complementary in charge [23]. Kyunguk Jeong first demonstrated that *Jug r 1* is a key marker for walnut allergy in very young children [24].

#### 2.1.2. *Jug r 2*

*Jug r 2* belongs to the 7S pea globulin family. It consists of 593 amino acids and is also one of the major allergens in walnuts, with a molecular weight of approximately 44 kDa [25]. Its structure exhibits a typical α-hairpin fold, stabilized by disulfide bonds formed by the conserved CxxxCxCxxxC motif. The C-terminal domain contains a β-barrel structure with a cupin domain [26,27], and the linear epitopes of *Jug r 2* are key factors in triggering allergic reactions to walnuts.

#### 2.1.3. *Jug r 3*

*Jug r 3* belongs to the non-specific lipid transfer protein (nsLTP) family. It consists of 119 amino acids with a molecular weight of 11.8 kDa. Its secondary structure comprises four α-helices, stabilized by four pairs of conserved disulfide bonds into a compact globular structure—a typical structural feature of non-specific lipid transfer protein type 1 (nsLTP1) family members. The disulfide bonds confer high structural stability, endowing the protein with heat- and digestion-resistant properties. The four α-helices enclose an internal hydrophobic cavity capable of accommodating various lipid ligands (fatty acids, phospholipids, etc.). Lipid binding influences local conformation, which in turn affects IgE recognition. Proteins in the nsLTP1 family generally exhibit extremely high thermal stability and are difficult to completely denature even through heat treatment [28].

#### 2.1.4. *Jug r 4*

*Jug r 4* is an allergen in walnuts belonging to the 11S legume globulin family. It consists of 507 amino acids and has a molecular weight of approximately 58 kDa [20]. 11S globulins typically assemble into hexameric complexes composed of six subunits (each pair consisting of an acidic and a basic subunit). They comprise N-terminal and C-terminal domains, forming a cupin fold, and exhibit significant sequence homology with globulin allergens from hazelnuts (*Cor a 9*) and cashews (*Ana o 2*). Robotham constructed a three-dimensional structural model of *Jug r 4* via homology modeling based on the atomic structure of soybean glycinin [29]. A recent study [30] used the SWISS-MODEL protein modeling software (https://www.swissmodel.expasy.org, accessed on 3 November 2024) to construct a structural model of *Jug r 4*, which was then employed for IgE epitope localization analysis.

#### 2.1.5. Others Walnut Allergenic Proteins

*Jug r 5* belongs to the PR-10 (pathogen-related protein 10) family and possesses the typical α-helix-β-sheet structural domains characteristic of this family; *Jug r 6* belongs to the 7S pea globulin (vicilin) subfamily of the cupin superfamily, with a monomeric molecular weight of approximately 47–50 kDa. In a European study, approximately 6% of walnut allergy patients were sensitive to *Jug r 6*. It exhibits moderate thermal stability and is highly susceptible to pepsin digestion. *Jug r 7* belongs to the actin-binding protein family (profilin). As a panallergen, it cross-reacts with profilins found in various pollens and plant-based foods, typically causing mild oral allergy symptoms. *Jug r 8* belongs to the non-specific lipid transfer protein type 2 (nsLTP2) family, with a molecular weight of 9 kDa; 4 out of 10 walnut-allergic subjects tested positive for IgE bound to this nsLTP2. *Jug r 9* belongs to Phospholipase D alpha 1, with a molecular weight of 92 kDa. Sera from 21 children who tested positive in an open oral food challenge with walnuts and in ImmunoCAP testing for walnuts and *Jug r 1* were analyzed. Of these, 5 (24%) had IgE to *Jug r 9.0101*, as determined by Enzyme-Linked Immunosorbent assay (ELISA). In addition to common walnuts, black walnuts also contain sensitizing proteins. *Jug n 1* is one of the major allergens in black walnuts; it is recognized by IgE in the serum of patients with walnut allergy. It belongs to the 2S albumin seed storage protein family and, similar to *Jug r 1*, is of significant value in the diagnosis of walnut allergy. *Jug n 2* belongs to the Vicilin seed storage protein; 7S globulin family. It is a highly stable, heat-resistant, and digestion-resistant Class I allergen, and conventional processing techniques cannot completely eliminate its allergenicity. Zhang first purified and identified *Jug n 4* from black walnuts [31]. Sodium dodecyl sulfate-polyacrylamide gel electrophoresis (SDS-PAGE) analysis revealed that it consists of subunits of 34 kDa and 22 kDa. *Jug n 4* belongs to the Legumin-like seed storage protein, a member of the 11S globulin family, with molecular weights of 34 or 22 kDa. Of 25 individuals with a history of tree nut allergy, eight showed positive IgE binding to the purified legumin in immunoblots performed under reducing conditions. The sensitization rate reached as high as 32%. The characteristics of walnut allergens are summarized in Table 1.

### 2.2. Techniques for Detecting Allergenic Proteins

**Figure 2:**
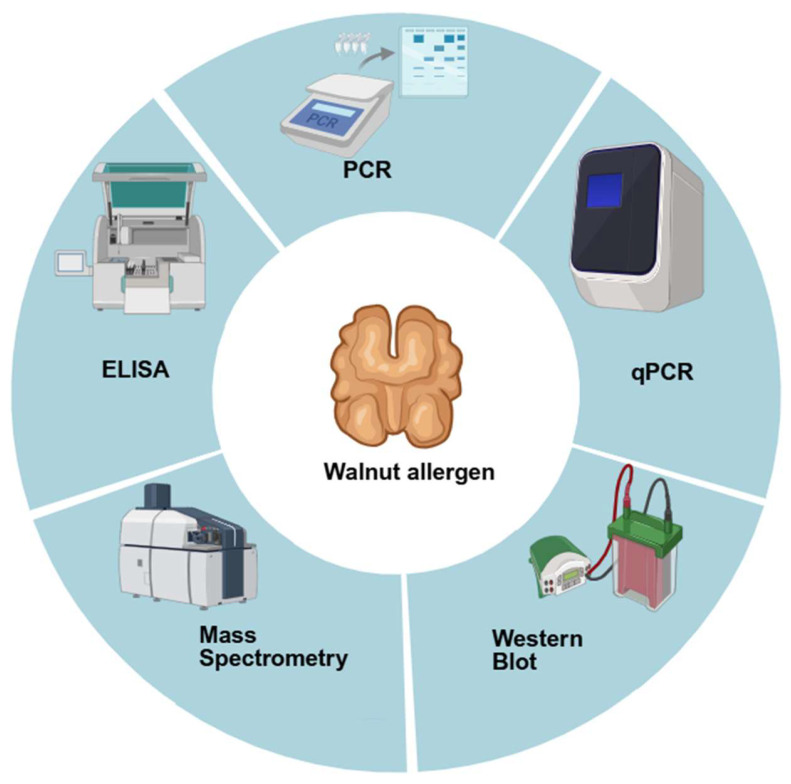
The Methods for Testing Walnut Allergens.

In recent years, there has been a rapid increase in demand for appropriate methods for detecting food allergens. The food industry has consistently paid close attention to the needs of consumers with food allergies and requires suitable analytical methods to detect allergenic proteins [32]. Since potential allergens in food typically exist in the form of specific proteins or characteristic DNA fragments and are present in low concentrations; therefore, there is a need to develop sensitive and effective methods for detecting allergens. In general, analytical methods for walnut testing can be divided into two categories: DNA-based methods and protein-based methods (Figure 2).

#### 2.2.1. DNA-Based Methods: Detection of Walnut-Derived Material

DNA-based methods, including conventional polymerase chain reaction (PCR), real-time PCR, and nested real-time PCR, are commonly used to determines whether a specific species or ingredient is present. PCR is a method based on the detection of DNA/RNA. It is highly specific, sensitive, simple, and rapid, though it requires relatively high sample purity. Currently, the most widely used methods for detecting walnut allergens are conventional PCR and real-time quantitative PCR. Conventional PCR is simple and time-efficient, requires minimal instrumentation, and offers high specificity [33]; Real-time PCR technology is a crucial tool for the rapid detection and quantification of food allergens. For example, a single-tube nested real-time fluorescent PCR system targeting the *Jug r 3* coding sequence can reduce the relative limit of detection for walnuts in dough and sponge cake matrices from 0.005% to 0.001%, with an absolute limit of detection as low as 1 pg of walnut DNA [34]. This technology not only represents a significant leap from qualitative to quantitative analysis but also, compared to conventional PCR, offers high specificity, effectively addresses PCR contamination issues, and features a high degree of automation. It is currently widely adopted. However, it can only detect foods containing ingredients derived from allergens, not the allergenic proteins themselves [35,36]. Therefore, a positive PCR result merely indicates the presence of walnut-derived material and does not necessarily reflect the content, integrity, or immunological activity of walnut allergens [37]. In addition, DNA degradation, PCR inhibitors, variations in DNA extraction efficiency, and the composition of the food matrix may all affect the reliability of the test, particularly in highly processed foods.

#### 2.2.2. Protein-Based Methods: Detection of Walnut Allergens

Protein-based methods, including ELISA, Western blotting, immunoblotting, and mass spectrometry, are more directly related to allergen detection because they target walnut proteins, allergenic epitopes, or allergen-specific peptides. The ELISA method can process a large number of samples continuously while maintaining a high level of sensitivity; it is a classic and practical method for detecting specific proteins. By utilizing specific antigen–antibody reactions, ELISA can detect walnut proteins or allergenic epitopes with high sensitivity and rapid readout. Currently, there are many studies on ELISA methods for walnut allergens, including non-competitive sandwich ELISA and indirect competitive ELISA, both of which have been successfully used to detect walnut allergens or their marker proteins in food matrices. The sandwich ELISA method developed for soluble walnut protein or 2S albumin has demonstrated good applicability in processed foods and has shown acceptable recovery rates and reproducibility in model food matrices. Interlaboratory validation studies using matrices such as cookies, bread, sponge cake, orange juice, jelly, chicken meatballs, and rice porridge demonstrated that the walnut ELISA kit yields reliable recovery rates and reproducibility, supporting its use for allergen labeling verification [38,39]. The primary advantage of immunological assays is their high specificity; they can accurately identify antigens and antibodies even in the presence of interfering background, thereby achieving the detection objective. Mass spectrometry (MS) overcomes the drawbacks of cross-reactivity in immunoassays and the inability of DNA-based techniques to directly detect allergenic proteins [40]. When combined with bioinformatics tools, mass spectrometry equipment can precisely identify, characterize, and quantify walnut allergenic proteins. In particular, liquid chromatography–tandem mass spectrometry (LC-MS/MS), Torii et al. developed an LC-MS/MS method for the simultaneous detection of walnut and almond allergens, demonstrating that precise detection is achievable even in complex processed food matrices [41].

#### 2.2.3. Method Validation and Limitations in Processed Foods

For the evaluation of walnut allergen detection methods, limit of detection (LOD), limit of quantification (LOQ), recovery, matrix effects, specificity, cross-reactivity, and interlaboratory reproducibility should be considered together. LOD reflects the lowest concentration of walnut-derived material, walnut protein, or allergen marker peptide that can be reliably detected, whereas LOQ represents the lowest concentration that can be quantified with acceptable accuracy and precision. Recovery is particularly important for walnut allergen analysis because walnut proteins may be difficult to extract completely from high-fat, high-polyphenol, heat-treated, or highly processed food matrices [34,38]. Food matrix effects may interfere with DNA extraction, PCR amplification, antibody recognition, protein extraction, enzymatic digestion, or peptide ionization in MS analysis. Therefore, method validation should not rely only on simple buffer systems or model samples, but should include representative processed foods such as bakery products, beverages, meat products, chocolate products, and mixed food matrices. Specificity and cross-reactivity should also be carefully assessed, especially because walnut allergens share structural similarities with allergens from closely related tree nuts such as pecan. In antibody-based assays, epitope masking, protein denaturation, aggregation, Maillard reactions, and polyphenol–protein interactions may lead to false-negative results, whereas cross-reactivity may result in false-positive signals. Interlaboratory validation is therefore necessary to confirm the reproducibility of a method across different laboratories, operators, instruments, and extraction protocols. Overall, we believe that DNA-based and protein-based methods are complementary; therefore, combined strategies using DNA-based and protein-based methods are recommended for complex and highly processed foods.

## 3. Recent Advances in Research on Food Processing and Allergenicity Modulation

**Figure 3:**
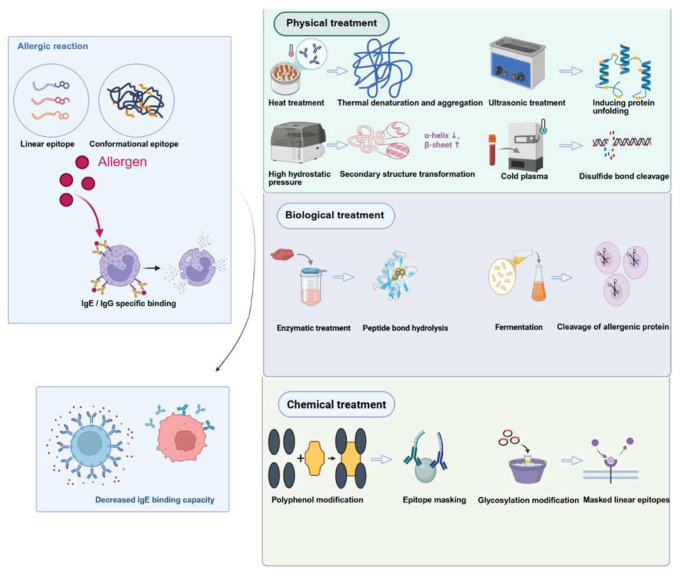
Impact of various processing technologies on allergenic proteins.

In recent years, there has been growing interest in using various food processing methods to reduce the allergenicity of walnuts. During food processing, the physical properties and chemical structures of food proteins often undergo changes. Factors such as processing methods and treatment intensity can influence the molecular characteristics of allergenic proteins. Structural changes may affect epitope accessibility and IgE/IgG binding in vitro, but do not necessarily predict clinical allergenicity [42]. Figure 3 illustrates the effects of different processing technologies on allergenic proteins.

Allergenic epitopes of allergenic proteins trigger allergic reactions in the body by binding to specific antibodies. Based on their structural characteristics, allergenic epitopes are classified into two types: linear epitopes and conformational epitopes. Linear epitopes consist of continuous amino acid sequences; conformational epitopes consist of amino acid sequences from different regions; these amino acids are structurally close to one another due to protein folding.

### 3.1. Distinguishing Structural Changes, Immunoreactivity, Digestibility, and Clinical Allergenicity

When considering the effects of processing on walnut allergens, it is important to distinguish between structural changes, immunoreactivity, digestibility, and clinical allergenicity. Although these concepts are related, they are not synonymous. Structural changes refer to alterations in the primary, secondary, tertiary, or quaternary structures of allergenic proteins, including denaturation, aggregation, hydrolysis, oxidation, glycosylation, or changes in epitope accessibility. Such changes do not directly prove that clinical allergenicity has been reduced. Immunoreactivity refers to the ability of an allergen or a processed allergen fragment to bind to antibodies, particularly IgE in allergic patients or IgG in immunological assays. A decrease in IgE/IgG binding capacity measured by ELISA, Western blotting, or inhibition assays indicates reduced antibody recognition in vitro under the test conditions. However, this does not necessarily imply a lower risk of allergic reactions in vivo. Digestibility refers to the susceptibility of a protein to gastrointestinal enzymes and simulated gastric or intestinal conditions [43]. Increased digestibility may contribute to allergen degradation. Incomplete digestion may release soluble immunoreactive fragments, and digestion-resistant proteins or peptides may still reach the intestinal immune system. Clinical allergenicity refers to the ability of a food or food-derived protein to induce allergic symptoms in sensitized individuals following exposure. It is influenced by factors such as the dose of the sensitizing agent, the food matrix, digestion, absorption, activation of effector cells, synergistic factors, individual sensitization characteristics, and medical history [44]. Therefore, clinical allergenicity cannot be fully predicted based solely on structural characterization or antibody binding assays.

Overall, structural changes, altered digestibility, reduced IgE/IgG binding, and clinical allergenicity represent different but interconnected levels of evidence. Structural characterization helps explain possible mechanisms, while IgE/IgG-binding assays and digestibility tests provide important in vitro information. However, these indicators alone cannot fully predict whether a processed walnut product will induce fewer allergic reactions in sensitized individuals. A reduction in clinical allergenicity may be inferred only when supported by in vivo or clinical evidence.

Table 2 summarizes the effects of different processing technologies on allergenic proteins. Based on their mechanisms of action and principles, these technologies can be classified into three major categories: physical methods, biological methods, and chemical methods. Physical methods may primarily alter conformational epitopes by disrupting the noncovalent bonds (such as hydrogen bonds, hydrophobic interactions, and disulfide bonds) that maintain the protein’s spatial conformation; biological methods may disrupt linear epitopes through proteolysis; whereas chemical methods could mask antigenic epitopes or alter protein conformation by forming covalent or noncovalent bonds with exogenous molecules.

### 3.2. Food Matrix Effects on Processing-Induced Changes in Walnut Protein Immunoreactivity

Food matrix effects should be considered when evaluating the influence of processing on walnut allergens. In many studies, walnut proteins have been investigated in the form of isolated protein extracts. However, in actual foods, walnut allergens are present in a complex matrix that may contain lipids, carbohydrates, polyphenols, salts, and other proteins [43]. These matrix components may interact with walnut allergenic proteins during processing, digestion, extraction, and immunoassay. Therefore, results obtained from purified walnut proteins or buffer-based model systems may not fully reflect the actual behavior of walnut allergens in processed foods [44].

Walnut kernels are rich in lipids. During heat treatment, ultrasonic treatment, high-pressure treatment, plasma treatment, or storage, lipids may affect protein solubility, aggregation, oxidation, and the formation of protein–lipid complexes. Lipid-rich matrices may also reduce protein extraction yields and alter the accessibility of IgE-binding epitopes. Furthermore, lipids may protect certain allergenic proteins from enzymatic degradation during digestion. These effects are particularly significant for allergens in walnuts, such as lipid transporters [28].

In baked goods, cereal products, confectionery, and mixed foods containing walnuts, reducing sugars can react with the amino groups of walnut proteins via the Maillard reaction during thermal processing. This process may mask IgE-binding epitopes and, in some cases, reduce antibody recognition [65,66]. However, it may also promote protein cross-linking, aggregation, the formation of advanced glycation end products (AGEs), or the generation of new epitopes. Therefore, the effect of the Maillard reaction on the immunoreactivity of walnut proteins depends on the type of sugar, reaction temperature, processing time, water activity, pH, food matrix composition, and the specific allergenic proteins involved [67].

The protective role of the food matrix during digestion is another important factor. Compared to isolated walnut protein, walnut allergens in whole food systems may be protected by lipids, carbohydrates, or other macromolecules [68]. This protective effect may delay digestion by pepsin or trypsin, thereby allowing larger sensitizing proteins or immunoreactive peptides to persist in the gastrointestinal tract [69,70]. Conversely, certain matrices may promote protein denaturation, thereby increasing the opportunity for enzymes to access them.

Food matrix effects also influence allergen detection and risk assessment. Processing-induced denaturation, aggregation, glycation, lipid oxidation, and protein–polyphenol interactions may reduce protein extraction efficiency or mask antibody recognition sites, leading to an underestimation of residual walnut proteins in ELISA or Western blot assays [71].

In summary, food matrix effects influence walnut allergen behavior during processing, digestion, extraction, detection, and immune recognition. Future studies should therefore evaluate walnut allergens not only as isolated proteins, but also in representative food matrices such as bakery products, chocolate products, beverages and mixed foods.

## 4. The Effect of Typical Processing Techniques on the Allergenicity of Walnut Protein

**Figure 4:**
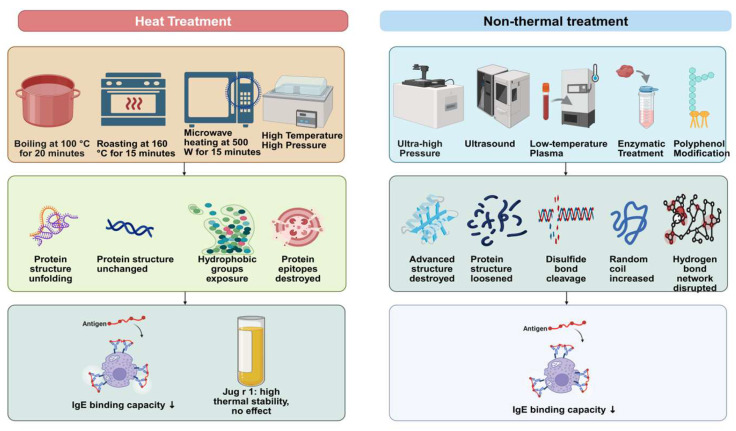
The Effect of Different Processing Technologies on the Allergenicity of Walnut Protein.

There are significant differences in the effects of various processing technologies on walnut protein, as shown in Figure 4.

### 4.1. Effects on the Structure of Walnut Proteins

Protein structure contributes to allergen recognition (primary, secondary, tertiary, and quaternary structures, as well as antigenic epitopes). The key mechanism by which processing techniques modulate IgE/IgG-binding capacity may involve modifying the chemical structure and spatial conformation of the proteins, thereby altering the exposure of antigenic epitopes and consequently affecting their ability to bind to IgE. There are significant differences in how various processing techniques affect the functional properties of walnut proteins.

#### 4.1.1. Effects of Heat Treatment on the Structure of Walnut Proteins

The primary effect of heat treatment is to cause protein denaturation and aggregation, inducing irreversible structural changes in walnut proteins. Essentially, this involves the breaking and restructuring of intra- and intermolecular chemical bonds (hydrogen bonds, disulfide bonds, and hydrophobic interactions), as well as the disruption and rearrangement of spatial conformation, thereby altering conformational epitopes. Different heat treatment methods have significantly different effects on protein structure: boiling at 100 °C for 20 min significantly unfolds the protein conformation; baking at 160 °C for 15 min does not cause significant disruption to the protein structure; microwaving at 500 W for 15 min exposes hydrophobic groups and causes slight aggregation; moist heat and high-temperature, high-pressure treatments cause more significant damage to protein structures (*p* < 0.05), leading to conformational unfolding and changes in epitope accessibility [72]. There are significant differences in the thermal stability of different walnut allergenic proteins. Bi Yuan et al. [73] found through thermal stability experiments that the *Jug r 1* protein exhibits extremely high thermal stability; after 60 min of heating, its primary, secondary, and tertiary structures remained intact, and the protein bands were preserved. Brown et al. [74] demonstrated that baking and heating had distinct effects on the structures of different allergen types: *Car i 2* and *Car i 4* (high molecular weight) unfolded and aggregated, while *Car i 4* (11S legumin) underwent rapid peptide chain cleavage and degradation (94% reduction), making it the most heat-sensitive; in contrast, *Car i 1* (2S albumin) remained structurally stable, with no significant changes observed in SDS-PAGE or Western blot bands after 24 min of heating. Since pecans and walnuts both belong to the Juglandaceae family, these findings provide important insights into the structural stability of walnut allergens.

#### 4.1.2. Effects of Non-Thermal Treatments on Walnut Protein Structure

##### Ultra-High Pressure (UHP) Technology

Ultra-high pressure (UHP) technology applies external pressures ranging from 100 to 1000 MPa to disrupt intramolecular and intermolecular non-covalent bonds (such as hydrogen bonds and hydrophobic interactions) in walnut proteins. This alters only the secondary and tertiary structures of the proteins (without disrupting the primary structure) and induces conformational rearrangement. Studies have shown that protein conformation does not undergo significant changes under low-temperature, high-pressure conditions (300–600 MPa) [75]. Jiang et al. [72] found that high-temperature, high-pressure treatment severely disrupted the original structure and antigenic epitopes of allergens in walnuts.

##### Ultrasonic Technology

Ultrasound modifies protein structure through two mechanisms: the cavitation effect and high-frequency shear forces. The former causes a sudden local increase in temperature and pressure, inducing conformational denaturation, while the latter disrupts hydrogen bonds between polypeptide chains and intermolecular forces, loosening the secondary and tertiary structures [76]. Zhang et al. [77] found that ultrasonic treatment at 400 W for 25 min disrupted the hydrogen bonds stabilizing α-helices, causing some α-helices to convert to β-sheets and β-turns, resulting in a looser protein structure. This structural change may reduce IgE recognition in vitro by altering the accessibility of conformational epitopes; however, processing may also expose previously hidden linear epitopes. Combined ultrasonic-microwave treatment (240 s, 90 °C, 700 W) can further weaken peptide bond interactions and alter spatial conformation [78]. Notably, peanut *Ara h 2* and walnut *Jug r 1* both belong to the 2S albumin family and share a highly similar structure. Fang et al. [79] found that ultrasonic treatment of peanut crude protein reduced the content of α-helices and caused a rearrangement of the protein’s spatial conformation. This result provides an important theoretical basis and methodological reference for allergenicity-reducing processing of walnuts.

##### Low-Temperature Plasma Technology

Low-temperature plasma induces the degradation and conformational rearrangement of walnut proteins by generating reactive free radicals [80]. Research by Deng et al. [81] indicates that the effect of treatment time on walnut protein structure follows a “first unfolding, then aggregation” pattern: at 90 s of treatment, the tertiary structure is fully unfolded, the secondary structure is most loosely packed (with the highest β-sheet content and lowest α-helix content), disulfide bonds are broken, free thiol groups increase, and conformational epitopes are destroyed; treatment exceeding 90 s leads to excessive oxidation, causing protein re-aggregation and a return to a denser structure. Further validation is needed regarding the specific structural effects of this technology on the major walnut allergens (*Jug r* 1–6).

##### Enzymatic Treatment

Enzymatic treatment disrupts the primary structure (linear epitopes) and spatial structure (conformational epitopes) of walnut proteins through hydrolysis, degrading them into small peptides and free amino acids. Limited proteolysis with trypsin increases the loosening of protein structures and the formation of random coils [82]; Alkaline proteases can degrade allergen precursor proteins such as *Jug r 4* and *Jug r 6*, causing their primary structures to break down and releasing peptide fragments. Dehulling promotes the unfolding of protein conformation, facilitating enzymatic hydrolysis [83]. Transglutaminase (TGase)-mediated cross-linking was utilized to alter the conformation of walnut allergenic proteins, reducing detectable immunoreactivity under experimental conditions [84,85]. However, enzymatic hydrolysis alone cannot completely destroy all IgE epitopes; it must be combined with techniques such as heat and pressure or ultrasound to synergistically disrupt protein structures. Combining instant controlled pressure-drop (DIC) with enzymatic hydrolysis can significantly disrupt protein structures; however, incomplete hydrolysis may result in the formation of immunoreactive peptides [86,87].

##### Polyphenol Modification

Polyphenols form complexes with walnut proteins through covalent or non-covalent interactions, inducing the restructuring of the protein’s secondary and tertiary structures [62]. Huang et al. [88] found that covalent binding of polyphenols EGCG, chlorogenic acid) to walnut-isolated proteins disrupts the original hydrogen bond network, alters the protein’s secondary and tertiary structures, and causes the protein conformation to unfold. Walnut skin polyphenols can bind to the most allergenic globulin, causing the globulin structure to unfold in a dose-dependent manner, with a reduction in α-helices and an increase in β-sheets and random coils, thereby disrupting conformational epitopes [89]. Covalent binding has a more significant effect on protein structure and can mask or alter epitope accessibility [90]. The binding of polyphenols may affect digestibility, sensory characteristics, and test results.

### 4.2. Effects of Typical Processing Technologies on Walnut Protein Function

Table 3 summarizes the mechanisms by which typical processing techniques affect the allergenicity of walnut protein, focusing on their technical principles, induced structural changes, functional consequences, key parameters. When assessing the impact of processing on walnut allergenicity, the evaluation is primarily based on research data specific to walnuts. Relevant evidence from other nuts or non-nut food allergens is limited to exploring potential mechanisms and cannot be directly extrapolated to indicate a reduction in the clinical allergenicity of walnuts.

### 4.3. Safety Considerations and Potential Risks of Processing-Induced Allergenicity Modulation

Although processing techniques hold promise for reducing the immunogenicity of walnut allergens, their effects are not always beneficial and cannot be directly equated with clinical safety. The impact of processing on allergenicity depends largely on the type of allergen, the intensity of processing, the food matrix, digestive conditions, and individual sensitization characteristics. This is particularly important for walnuts, as some of their major allergens exhibit high structural stability. For example, *Jug r 1* is a 2S albumin with a well-defined IgE-binding epitope that exhibits strong resistance to both thermal denaturation and enzymatic digestion; meanwhile, *Jug r 3* is a non-specific lipid transfer protein (nsLTP) stabilized by conserved disulfide bonds, which also possesses good thermal and digestive stability. A study on processed walnuts showed that significant amounts of IgE-binding proteins could still be detected after dry roasting; even though boiling significantly reduced IgE-binding proteins, proteins such as *Jug r 1* and *Jug r 3* remained stable after processing [92].

First, processing may expose hidden epitopes or generate new immunoreactive structures. Techniques such as heat treatment, sonication, high-pressure treatment, plasma treatment, enzymatic hydrolysis, and polyphenol modification can denature proteins, thereby destroying conformational epitopes. However, protein unfolding may also expose buried linear epitopes that were previously inaccessible to IgE antibodies. This is particularly evident in physical treatments such as sonication and high-pressure processing, as these methods primarily alter the protein’s secondary, tertiary, or quaternary structure while often preserving the original amino acid sequence. Therefore, although conformational epitopes may be reduced, linear epitopes may remain intact or become more readily recognizable.

Second, enzymatic treatment can directly cleave peptide bonds and disrupt linear epitopes, which is one of the most effective strategies for reducing allergenicity. However, incomplete hydrolysis may result in the formation of low-molecular-weight peptides that retain sequences capable of binding to IgE or eliciting T-cell responses. Improved digestibility does not always translate to a reduced risk of allergy, as the digestive process under gastrointestinal conditions may also release soluble immunoreactive fragments. Therefore, safety assessments of enzymatically treated walnut proteins should include peptide size distribution, residual IgE-binding capacity, and simulated gastrointestinal digestion tests.

Third, glycation may reduce IgE recognition by masking sensitizing epitopes; however, the Maillard reaction can also generate advanced glycation end products, cross-linked aggregates, or novel epitopes. The ultimate effect of Maillard reaction products on sensitization varies and depends on the type of protein, the type of sugar, the stage of the reaction, processing temperature, processing time, and the food matrix [65,66,67].

Fourth, certain processing conditions may have a negative impact on nutritional and sensory quality. Although intense heat and pressure treatment or severe oxidation can reduce IgE/IgG binding, these processes may also lead to protein aggregation, amino acid oxidation, reduced solubility, the formation of off-flavors, and the deterioration of functional properties. Although polyphenol modification can enhance antioxidant activity and mask epitopes, excessive protein–polyphenol interactions may alter astringency, color, digestibility, and consumer acceptance. Therefore, the development of hypoallergenic walnut products must be achieved while maintaining nutritional value, sensory acceptability, and technical feasibility.

In general, when evaluating the effectiveness of processing in reducing allergenicity, one should simultaneously consider the potential exposure of hidden epitopes, the formation of immunoreactive peptide fragments, Maillard reaction products, nutrient loss, deterioration in sensory quality, and matrix effects. Relying solely on in vitro IgE/IgG binding assays, ELISA, Western blotting, or structural characterization is far from sufficient. Future research should integrate molecular characterization, validated allergen detection methods, simulated digestion models, cellular assays, nutritional and sensory evaluations, and animal models, only then can the actual allergenic risk of processed walnut products be assessed.

## 5. Current Status of the Development and Application of Low-Allergenic Foods

Food allergies have become a global public health issue, affecting the quality of life for hundreds of millions of people. Low-allergenic foods refer to foods that have undergone physical, chemical, or biotechnological processing to reduce their in vitro immunoreactivity. Currently, there is no cure for food allergies, and strictly avoiding allergenic foods remains the only effective management strategy. However, due to the widespread presence of allergens and the risk of cross-contamination, complete avoidance is difficult to achieve. Therefore, the development of low-allergenic foods has emerged as a key approach to addressing this challenge, aiming to reduce or eliminate food allergenicity through processing technologies and may provide future dietary options after safety validation for people with allergies. Currently, most research on the principles of hypoallergenic food preparation remains at the whole-protein level; future efforts should focus more on technologies that reduce allergenicity by targeting allergen epitopes [93].

Previous studies [94] have confirmed that the addition of bayberry leaf (BBL) can reduce IgE-binding capacity or antigenicity under tested conditions of bread. El-Aziz and colleagues investigated three hypoallergenic infant formula formulations (chickpea–rice, corn–egg white, and potato–mushroom) and analyzed their nutritional components, amino acid profiles, vitamin content, and antioxidant activity, while conducting sensory evaluations to provide a basis for the industrial production of hypoallergenic infant foods. Arteaga-Marin et al. [18] noted that while non-thermal processing technologies can reduce the immunogenicity of plant proteins by more than 50%, the degree of desensitization varies among individuals. The negative impact of processing on the nutritional value and functional properties of proteins, as well as the lack of a standardized low-allergenicity evaluation system, are the primary challenges currently faced. Pang et al. [95] believe that combined processing technologies represent the mainstream direction for the development of hypoallergenic foods, and that the combination of physical and biochemical methods can produce synergistic effects, yielding more significant results than single technologies. Narciso et al. pointed out that non-thermal treatments such as microwaves, high pressure, and ultrasound can serve as pretreatments for further chemical or biochemical processing, providing new insights for precise allergen reduction [17].

From an industrial perspective, the development of allergenicity-reduced walnut products should be treated cautiously. Although several processing technologies, including high-temperature and high-pressure treatment, enzymatic hydrolysis, polyphenol modification, and combined processing strategies, have shown potential for reducing walnut protein immunoreactivity, most available evidence is still based on laboratory-scale experiments, purified protein systems, or model food matrices [44]. Before industrial application, several issues need to be systematically addressed. First, processing parameters should be optimized and validated under scale-up conditions to ensure batch-to-batch reproducibility. Second, the residual immunoreactivity of processed walnut proteins should be evaluated in representative food matrices rather than only in buffer systems or isolated protein extracts. Third, the effects of processing on nutritional quality, sensory properties, texture, color, flavor, storage stability, and consumer acceptance should be assessed. Fourth, reliable and validated analytical methods are required to quantify residual walnut proteins or marker peptides in complex processed foods.

## 6. Conclusions

The walnut is a nutritionally valuable tree nut, but its allergenicity remains a major concern for sensitive consumers. The major walnut allergens, especially *Jug r 1*, *Jug r 2*, *Jug r 3*, and *Jug r 4*, are clinically important because they contain IgE-binding epitopes and show different degrees of resistance to heat, digestion, and food processing. Therefore, understanding the molecular characteristics, epitope distribution, stability, and cross-reactivity of these allergens is essential for walnut allergy diagnosis, allergen detection, and the development of allergenicity-reducing strategies.

Current evidence suggests that various processing techniques can reduce the immunoreactivity of walnut proteins by altering protein conformation, disrupting linear or conformational epitopes, or masking antigenic recognition sites. Among the existing strategies, high-temperature and high-pressure treatment, enzymatic hydrolysis, polyphenol modification, and integrated processing methods appear to hold promise. In particular, combined methods such as heat–pressure-assisted enzymatic hydrolysis, ultrasound-assisted enzymatic hydrolysis, and polyphenol-related modifications may yield more significant results than single-treatment methods, as they act on walnut proteins through complementary mechanisms.

However, the current evidence remains insufficient to support overly optimistic conclusions. Most studies are still based on in vitro assays, such as IgE/IgG binding, ELISA, Western blotting, or structural characterization. These methods are useful for evaluating changes in immunoreactivity, but they cannot fully predict clinical tolerance in patients with walnut allergies. In addition, processing may expose hidden epitopes, generate immunoreactive peptide fragments, induce Maillard reaction products, or negatively affect nutritional and sensory quality. Therefore, reduced in vitro immunoreactivity should not be directly interpreted as clinical safety.

Future research should shift from descriptive observations to mechanism-based and clinically meaningful assessments. Priority should be given to establishing the relationship between structure, epitopes, and allergenicity; identifying processing parameters for major walnut allergens that do not compromise nutritional or sensory quality; and developing a standardized evaluation system that integrates molecular characterization, validated allergen detection, simulated digestion, cellular assays, animal models, and clinical validation. In terms of industrial translation, technologies aimed at reducing allergenicity should be evaluated by comprehensively considering product quality, cost, regulatory requirements, labeling, and consumer acceptance.

## Figures and Tables

**Table 1:** The characteristics of walnut allergens.

Allergen	Biochemical Name	MW(SDS-PAGE)	Isomer	Nucleotide Sequence (NCBI)	Protein Sequence (NCBI)	Protein Sequence (UniProt)
*Jug r 1*	2S albumin seed storage protein	15–16 kDa	*Jug r* 1.0101	U66866	AAB431O8	P93198
*Jug r 2*	Vicilin (7S globulin) seed storage protein containing N-terminal alpha-hairpinin peptides	44 kDa	*Jug r* 2.0101 *Jug r* 2.0101 (1–173) *Jug r* 2.0102 *Jug r* 2.0102 (27–367) *Jug r* 2.0102 (368–789)	AF066055 AFO66O55 (1–519) XM-018956626	AAF18269 AAF18269 (1–173) XP018812171 XP-018812171 (27–367) XP-018812171 (368–789)	Q9SEW4 Q9SEW4 (1–173) A0A2I4DYF1 A0A2I4DYF1 (27–367) A0A2I4DYF1 (368–789)
*Jug r 3*	Non-specific lipid transfer protein type 1 (nsLTP1)	9 kDa	*Jug r* 3.0101	EU780670	ACI47547	C5H617
*Jug r 4*	11S globulin seed storage protein	58.1 kDa	*Jug r* 4.0101	AY692446	AAW29810	Q2TPW5
*Jug r 5*	PR-10	20 kDa	*Jug r* 5.0101	KX034087.1	APD76154.1	
*Jug r 6*	7S globulin seed storage protein, vicilin-like protein	47 kDa	*Jug r* 6.0101	XM-018959147	XP-018814692	A0A2I4E5L6
*Jug r 7*	Profilin	13 kDa	*Jug r* 7.0101	MG366484	AVD53651	A0A2I4DNN6
*Jug r 8*	Non-specific lipid transfer protein type 2 (nsLTP2)	9 kDa	*Jug r* 8.0101 *Jug r* 8.0201	XM-018961116 XM-018991569	XP-018816661 XP-018847114	A0A2I4EB91 A0A2I4GT96
*Jug r 9*	Phospholipase D alpha 1	92 kDa	*Jug r* 9.0101	XM-018996162	XP-018851707	A0A2I4H6D4
*Jug n 1*	2S albumin seed storage protein		*Jug n* 1.0101	AY102930	AAM54365	Q7Y1C2
*Jug n 2*	Vicilin seed storage protein; 7S globulin		*Jug n* 2.0101	AY102931	AAM54366	Q7Y1C1
*Jug n 4*	Legumin-like seed storage protein; 11S globulin	34 kDa, 22 kDa	*Jug n* 4.0101	KX891230	APR62629	A0A1L6K371

**Table 2:** The effects of different processing technologies on allergenic proteins.

Processing Technology	Technical Principles	Allergenic Foods or Proteins	Changes in IgE Binding or In Vitro Immunoreactivity	Technical Specifications	References
Heat treatment	Disruption of hydrogen bonds, disulfide bonds, and hydrophobic interactions leads to protein denaturation and aggregation	Peanut allergen (*Ara h 1*)	Reduced IgE binding efficiency	Boil at 100 °C for 20 min	[45]
		Eggs	Reduced IgE binding or antigenicity in vitro	Heat at 80 °C for 10 min	[44,46]
Ultra-high pressure	Break non-covalent bonds (hydrogen bonds, hydrophobic interactions) and alter tertiary and quaternary structures	soybeans	Reduced in vitro immunoreactivity	300 MPa, germination after 15 min	[47]
		peach	The IgE-binding capacity of peach protein is significantly reduced	600 Mpa	[48]
		ovalbumin	Significantly reduces ovalbumin in vitro immunoreactivity	600 Mpa	[49]
Ultrasound	The cavitation effect disrupts hydrogen bonds and intermolecular forces; shear forces cause protein depolymerization	soybeans	When allergens are broken down into peptides and amino acids, the IgE binding of soybean sprout protein decreases significantly.	300 W	[50]
		Anisyl protein	Markedly reduced IgE-binding capacity under tested conditions	300 W ultrasonic transducer	[51]
		Shrimp myosin	Potentially reduce allergenic risk, with the secondary structure transitioning from random coils to β-turns/β-sheets	300 W, 20 kHz	[17,52]
		Kiwi fruit protein (*Act d 2*)	*Act D2* levels reduced by 50%	400 W, 16 min	[53]
Low-temperature plasma	Oxidative modification by reactive oxygen/nitrogen species; cleavage of disulfide bonds; induced cross-linking	Peanut (*Ara h 1*)	Antigenicity was reduced by approximately 55%	13 min	[54]
		Milk casein	The linear epitope is disrupted, significantly reducing the antigenicity of casein.	Argon plasma, 12 min	[55]
		Shrimp myosin	α-helix ↓69%, β-sheet ↑36%, IgE-binding capacity ↓96%	20 min	[56]
Enzyme treatment	Hydrolyze peptide bonds, disrupting linear and conformational epitopes	Rapeseed Bee Pollen	A significant decrease in IgE-binding capacity and a significant increase in amino acid and oligopeptide content	Enzyme blend (pectinase + cellulase + papain)	[57]
		Almond milk	The linear epitope of the bitter almond allergen was reduced by 57.14%	Papain	[58]
Fermentation	Cleave the allergen into smaller peptide fragments or amino acids, thereby destroying its key linear antigenic epitopes	Cow’s milk αs1-casein	The antigen inhibition rate was 72.27%, and there was a significant increase in the types and content of free amino acids.	*Lactobacillus plantarum JY067* fermented for 27 h	[59]
		Soy protein isolate	A significant decrease in IgE-binding capacity, with antigenic epitopes being hydrolyzed and destroyed	*Lactobacillus plantarum*	[60]
Polyphenol modification	It forms covalent or non-covalent bonds with the amino acid side chains of the allergen, altering the allergen’s spatial conformation and thereby modifying or masking the antigenic epitopes.	β-lactoglobulin (β-LG)	Significantly reduced IgE-binding capacity	Covalent binding of epigallocatechin gallate (EGCG) and chlorogenic acid (CA)	[61]
		soybeans	Reduce immunoreactivity, improved functional properties	Interactions between Polyphenols and Soy Protein	[62]
Glycosylation	Masking linear epitopes via the Maillard reaction to reduce immunoglobulin E recognition	Macadamia nuts	Reduced in vitro immunoreactivity of macadamia protein	Glucose/Sucrose Dry Sugar Glycosylation	[63]
		Wheat	Reduced IgE binding to γ-globulin and ω1,2-globulin	Dry glycosylation	[64]
		Shrimp myosin	Unfolding of the (tropomyosin) TM primary structure, alteration of the α-helix structure, and reduced IgE-binding capacity	Oligosaccharide glycosylation	[59]

**Table 3:** Mechanisms by Which Typical Processing Techniques Affect the Allergenicity of Walnut Protein.

Processing Technology	Technical Principles	Structural Changes in Walnut Protein	Functional Changes in Walnut Protein	Key Parameters	References
Heat treatment	Breaking hydrogen bonds, disulfide bonds, and hydrophobic interactions	*Jug r 1* is structurally stable; *Jug r 4* is structurally disrupted; severe conformational unfolding caused by high temperature and pressure	Humid heat/high temperature and high pressure: Significant decrease in IgE/IgG binding capacity (*p* < 0.05); *Jug r 1* binding capacity remains unchanged	Boil at 100 °C for 20 min; high temperature and high pressure: 256 kPa, 138 °C	[72,73,74]
Ultra-high pressure	Break non-covalent bonds and induce conformational rearrangement	Low temperature and high pressure: no significant change in conformation; high temperature and high pressure: severe structural damage	Low temperature and high pressure: No significant change in function; High temperature and high pressure: Significant decrease in IgE binding capacity	300–600 MPa; high temperature and high pressure: 256 kPa, 138 °C	[72,75,91]
Ultrasound	Cavitation effect + high-frequency shear forces	α-helix ↓, β-sheet ↑; primary structure intact	Reduced IgE recognition of conformational epitopes; preservation of linear epitopes; improved functional properties	400 W, 25 min	[76,77,78]
Low-temperature plasma	Reactive radical-induced degradation, conformational rearrangement	<90 s: Most loosely structured; >90 s: Reaggregation	90 s: Optimal performance; >90 s: Performance ↓	90 s	[80,81]
Enzyme treatment	Hydrolysis breaks peptide bonds	Primary structural break; loose structure, irregular curling ↑	Solubility ↑; IgE-binding capacity ↓; incomplete hydrolysis by a single enzyme	Trypsin/Alkaline protease	[82,83,86,87]
Polyphenol modification	Covalent/non-covalent binding, inducing structural rearrangement	α-helix ↓, β-sheet ↑; structural unfolding	Solubility ↑, antioxidant activity ↑; IgE/IgG binding capacity ↓	20% addition, pH 7	[62,88,89,90]

## Data Availability

No new data were created or analyzed in this study. Data sharing is not applicable to this article.

## Abbreviations

The following abbreviations are used in this manuscript:

FDA	Food and Drug Administration
IgE	Immunoglobulin E
nsLTP	non-specific lipid transfer protein
PR-10	Pathogen-related protein 10
nsLTP2	non-specific lipid transfer protein type 2
PCR	Polymerase Chain Reaction
WB	Western blot
ELISA	enzyme-linked immunosorbent assay
MS	Mass spectrometry
LC-MS/MS	liquid chromatography–tandem mass spectrometry
IgG	Immunoglobulin G
UHP	Ultra-high pressure
HTHP	High-temperature, high-pressure
AGEs	advanced glycation end products
BBL	bayberry leaf
CA	chlorogenic acid
DIC	instant controlled pressure-drop
ED	eliciting dose
EGCG	epigallocatechin gallate
LOD	limit of detection
LOQ	limit of quantification
SDS-PAGE	sodium dodecyl sulfate–polyacrylamide gel electrophoresis
TM	tropomyosin
β-LG	β-lactoglobulin
